# Molecular and Ecological Studies of a Virus Family (*Iridoviridae*) Infecting Invertebrates and Ectothermic Vertebrates

**DOI:** 10.3390/v11060538

**Published:** 2019-06-09

**Authors:** V. Gregory Chinchar, Amanda L. J. Duffus

**Affiliations:** 1 Department of Microbiology and Immunology, University of Mississippi Medical Center, Jackson, MS 39216, USA; 2 Department of Mathematics and Natural Sciences, Gordon State College, Barnesville, GA 30204, USA; aduffus@gordonstate.edu

Research involving viruses within the family *Iridoviridae* (generically designated iridovirids to distinguish members of the family *Iridoviridae* from members of the genus *Iridovirus*) has markedly increased in recent years. Inspection of data from PubMed indicates that from 1990 to 1999 approximately 60 articles related to this family appeared in the literature, whereas over 850 articles involving various iridovirids were published between 2010 and 2019. The marked upsurge in publications reflects the fact that iridovirids, once viewed as obscure viruses with little economic or ecological impact, are now known to be widely distributed in nature, infect a large and diverse array of invertebrates and ectothermic vertebrates, and trigger marked levels of morbidity and mortality in specific populations (e.g., endangered or commercially-important species) [1].

Currently, six genera comprise the family *Iridoviridae*: three which infect invertebrates (*Iridovirus*, *Chloriridovirus*, and *Decapodiridovirus*), two that target only bony fish (*Lymphocystivirus* and *Megalocytivirus*), and one that infects fish, amphibians, and reptiles (*Ranavirus*) [2]. Lymphocystis disease has been recognized for over a century among marine and freshwater fish species. However, this clinical presentation was not formally linked to a virus until the 1960s and the inability to propagate the virus easily in cell culture markedly impeded its study [3]. Invertebrate iridescent viruses (IIV) were identified in the mid-1950s and subsequently shown to infect a large number of invertebrate species [4,5,6]. However, for a variety of reasons (e.g., the absence of significant economic or ecological impact, the paucity of robust *in vitro* systems), their study has not progressed along with those of their vertebrate virus counterparts. In contrast, the identification of *Frog virus 3* (FV3, genus *Ranavirus*) from North American leopard frogs (*Lithobates pipiens*) in 1965 by Granoff and coworkers [7] led to the characterization of the family at the molecular level and the identification of a number of characteristic features including a circularly permuted and terminally redundant genome, rapid turnoff of host protein and RNA synthesis triggered by a virion-associated protein, and, among vertebrate viruses, methylation of cytosine residues within the sequence CpG [8]. Moreover, phylogenetic analysis of the complete genomic sequences of over 40 viruses has solidified our understanding of iridovirid taxonomy and has indicated relatedness to other large DNA containing viruses such as ascovirus, mimivirus, and marseillevirus. Recently phylogenetic analysis of isolates from shrimp and crayfish led to the establishment of a third genus (*Decapodiridovirus*) within the subfamily *Betairidovirinae*, and studies of fish and reptiles suggest the possible existence of a fourth genus within the *Alphairidovirinae* encompassing erythrocytic necrosis viruses [9,10,11,12,13].

Morphologically, iridovirids are large, icosahedral double-stranded DNA-containing viruses containing a DNA-protein core surrounded by an internal lipid membrane, an icosahedral protein capsid, and, in those viruses released by budding, a viral envelope that may also display a fringe of fibrils [14]. Viral genomes range in size from ~100–200 kbp and encode between 100 and 200 putative proteins. Replication involves both the nucleus and cytoplasm. Early viral transcription and 1st stage DNA synthesis take place within the nucleus, whereas late viral transcription and 2^nd^ stage DNA synthesis (concatemer formation) take place in the cytoplasm. Virions assemble within morphologically distinct cytoplasmic assembly sites and are released either by cell lysis or by budding from the plasma membrane [14,15,16,17].

In this issue of *Viruses*, we provide a collection of articles focused on two different aspects of iridovirid biology: the ecology of iridovirus infections and studies using molecular, immunological, and phylogenetic tools to understand the roles of iridovirid proteins, the interaction between iridoviruses and the host immune system, and the taxonomic relationships among members of the family *Iridoviridae*, nuclear cytoplasmic large DNA viruses (e.g., poxvirus, ascovirus, phycodnavirus, asfivirus), and the newly-identified “Giant Viruses” (e.g., marseillevirus, mimivirus, etc.). Ecological studies have focused on identifying new viral species and hosts, characterizing the pathological outcomes of infection, ascertaining how environmental influences impact the severity of infection, developing models to explain virus spread and disease, and understanding the consequences of infections among wild and cultured species. Molecular and phylogenetic studies center on identifying and determining the function of essential viral replicative genes required for growth both *in vivo* and *in vitro*, and in identifying and determining the function of virus-encoded “immune evasion” and “efficiency genes.” These latter genes, although not required for replication in cell culture, are absolutely required for replication *in vivo*. Immune evasion genes function by inhibiting innate and acquired anti-viral responses, whereas efficiency genes permit replication under restrictive cellular environments (e.g., low nucleotide pool levels). Lastly immunological studies attempt to define the elements of the host immune response required to provide a protective response. Collectively, articles found within this issue of *Viruses* provide a snapshot of ongoing studies in the field.
